# Differential Grey Matter Changes in Sensorimotor Cortex Related to Exceptional Fine Motor Skills

**DOI:** 10.1371/journal.pone.0051900

**Published:** 2012-12-26

**Authors:** M. Cornelia Stoeckel, Farina Morgenroth, Cathrin M. Buetefisch, Rüdiger J. Seitz

**Affiliations:** 1 Department of Systems Neuroscience, University Medical Center Hamburg-Eppendorf, Hamburg, Germany; 2 Department of Neurology, University Hospital Düsseldorf, Düsseldorf, Germany; 3 Departments of Neurology and Rehabilitation Medicine, Emory University, Atlanta, Georgia, United States of America; 4 Biomedical Research Centre, Heinrich-Heine-University, Düsseldorf, Germany; 5 Brain Imaging Centre West, Jülich, Germany; University of Reading, United Kingdom

## Abstract

Functional changes in sensorimotor representation occur in response to use and lesion throughout life. Emerging evidence suggests that functional changes are paralleled by respective macroscopic structural changes. In the present study we used voxel-based morphometry to investigate sensorimotor cortex in subjects with congenitally malformed upper extremities. We expected increased or decreased grey matter to parallel the enlarged or reduced functional representations we reported previously. More specifically, we expected decreased grey matter values in lateral sensorimotor cortex related to compromised hand function and increased grey matter values in medial sensorimotor cortex due to compensatory foot use. We found a medial cluster of grey matter increase in subjects with frequent, hand-like compensatory foot use. This increase was predominantly seen for lateral premotor, supplementary motor, and motor areas and only marginally involved somatosensory cortex. Contrary to our expectation, subjects with a reduced number of fingers, who had shown shrinkage of the functional hand representation previously, did not show decreased grey matter values within lateral sensorimotor cortex. Our data suggest that functional plastic changes in sensorimotor cortex can be associated with increases in grey matter but may also occur in otherwise macroscopically normal appearing grey matter volumes. Furthermore, macroscopic structural changes in motor and premotor areas may be observed without respective changes in somatosensory cortex.

## Introduction

Life-long plasticity is one of the most fascinating features of the human brain. It is furthermore holding promise for functional compensation and repair following injury acquired early or later in life. The capacity for functional reorganization was first shown for the non-human brain [1], [2]. However, the appearance of non-invasive neuroimaging technology brought evidence for functional plasticity due to learning and adaptation in the healthy and the lesioned developing and mature human brain. Evidence for functional reorganization was followed by studies demonstrating similar macroscopic structural changes of the cerebral cortex. Gaser and Schlaug (2003) demonstrated specific differences in brain structure between professional musicians and non-musicians [3]. Later it was demonstrated, that the training of juggling over a period of only three months resulted in the emergence of more grey matter in areas that are involved in the perception and processing of complex visual motion [4]. Even more recently, changes in grey and white matter were demonstrated after just 6 weeks of juggling [5]. Functional changes in chronic pain patients were paralleled by structural changes [6] and reversible within 12–18 weeks once patients are adequately treated [7]. Grey- or white-matter changes in humans were usually based on group differences in local values in MRI-scans as discovered by voxel-based morphometry (VBM). Up to now only animal studies allow the investigation of the anatomical substrate of such “structural” changes as discussed elsewhere [2].

Surprisingly, studies demonstrating corresponding functional and structural changes in the same group of individuals are rare [8], [9]. In the present study we re-investigated data acquired previously to test the hypothesis that functional changes we had observed in subjects with congenitally malformed upper extremities and compensatory skilful foot use [10]–[13] would be paralleled by structural changes of sensorimotor cortex. Our previous findings comprised a reduced somatosensory hand representation that was related to the number of missing fingers [11], [12]. In contrast, subjects with compensatory foot use showed an enhanced representation of the foot in medial somatosensory cortex together with superior stimulus localization on the toes when compared to age matched controls [10]. For primary motor cortex the most striking finding in subjects with frequent, hand-like compensatory foot use was an abnormal somatotopy where the foot was represented in two non- adjacent areas of motor cortex. One representation was located in the classical medial foot area. The second representation was located several centimetres away from the classical foot representation in the vicinity of the lateral ‘hand’ area [13]. Taken together, these results demonstrate a markedly abnormal functional organization of upper and lower limb representations in sensorimotor cortex.

In the present study we wanted to determine whether the particular functional organization of our subjects was associated with similar abnormalities in structural organization. We used VBM of T1-weighted MR images to compare the macroscopic morphology of the sensorimotor cortex in our subjects with malformed upper extremities to age-matched control subjects. More specifically, we assumed that there would be no general effect related to the cause of the malformation (intra-uterine thalidomide-exposure) but a specific effect related to malformation and compensatory foot use. We hypothesized increased grey matter values in the sensorimotor foot representation of subjects showing frequent, hand-like compensatory foot use in every-day life as the functional foot representation was increased in our previous studies [10], [13]. Furthermore, we expected decreased grey matter values for the hand area of subjects with upper-extremity malformations, who had previously been shown to have a reduced functional hand representation [11], [12].

## Materials and Methods

### Ethics Statement

The study was approved by the Ethics Committee of the Heinrich-Heine University of Düsseldorf. All subjects gave informed written consent prior to scanning and the whole study was conducted in accordance with the principles expressed in the Declaration of Helsinki.

### Subjects

**Figure 1:**
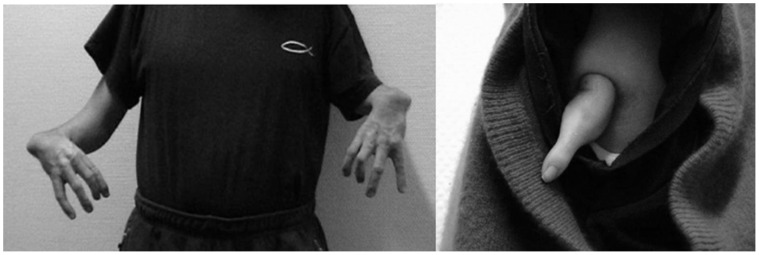
Upper-extremity dysmelia. Subject T2-5 shows bilateral dysmelic malformations with missing thumbs on both sides and symmetric foreshortening of both arms. The particular shape of the arms is due to bilaterally missing humeri (A). The residual finger in subjects T3-2 is attached directly to the shoulder (B).

In all subjects upper-extremity malformation presented as upper extremity dysmelia due to intra-uterine thalidomide exposure. The sedative thalidomide was recently back in the headlines when the molecular basis of its teratogenicity was unravelled [14]. About 50 years ago this agent caused severe birth defects when it was prescribed to pregnant women during the first trimester. Depending on the exact timing of exposure during gestation thalidomide can cause damage to upper and lower extremities, ears, cranial nerves and internal organs [15]. Of these abnormalities, upper-extremity dysmelia is the most striking effect of intra-uterine thalidomide-exposure. This so-called dysmelic syndrome has been described in details elsewhere [16]. In short, malformations follow a particular pattern: First, fingers fail development in a systematic way. If four fingers are developed the thumb is missing (Figure 1A). If only one finger is developed, the remaining finger corresponds to the little finger (Figure 1B). Second, foreshortening of the arms is positively correlated with the number of missing fingers. Third, the pattern of the malformation is highly symmetric (Figure 1A), generally resulting in bilateral malformations. Subjects lacking only one finger, i.e. the thumb, have already considerably compromised hand-function as the thumb is central to the opposition grasp. Subjects with severely foreshortened arms are not able to perform bilateral object manipulation with the hands. Depending on the degree of the malformation, hand function is compromised and forces subjects to use their feet for activities of daily living. Compensatory foot use is therefore highly dependent on compromised hand function, which in turn depends on the degree of the malformation. Furthermore foot use depends on whether subjects were encouraged or discouraged to use their feet during childhood development. Therefore, not all thalidomide-exposed subjects with compromised hand function are foot users.

According to our hypotheses the effect of three subject characteristics (variables) on grey matter volume was tested using VBM: (1) prenatal thalidomide exposure (yes, no), (2) compromised hand function (yes, no) and (3) foot use (no, occasional, frequent and hand like). For the variable “foot use” a categorical distinction between groups seemed appropriate because foot use was qualitatively and quantitatively different. Subjects with occasional foot use used one foot for actions such as “picking up objects” while subjects with frequent, hand-like foot use used their feet for all activities of daily living including highly skilled activities such as knitting or typing on a key board.

Because the variables were not independent, it was not possible to investigate them within a factorial design (see below).

Data were obtained from 40 subjects with no previous history of neurological or psychiatric disease. Subjects were assigned to the following 5 different groups according to the three above mentioned variables: T1: Thalidomide exposure, compromised hand function, *no foot use*; T2: Thalidomide exposure, compromised hand function, *occasional foot use*; T3: Thalidomide exposure, compromised hand function, *frequent, hand-like foot use*; TC: Thalidomide exposure, normal hand function, no foot use (this group served as a thalidomide-exposed control group with intact hand function and no compensatory foot use); C: No thalidomide exposure, normal hand function, no foot use (this group served as a control for thalidomide exposure).

Out of 30 thalidomide-exposed subjects, 20 subjects showed upper-extremity malformations with restrictions of hand function. Their malformations ranged from only slightly foreshortened arms and one missing finger, i.e. the thumb, to complete bilateral amelia. Detailed information on malformations and self-reported foot use for groups T1–T3 is summarized in Table 1. The remaining 10 subjects with thalidomide-exposure but no restriction of hand function were allocated to group TC. Thalidomide-exposed subjects were compared to 10 age-matched subjects with no exposure to thalidomide (C). Group-sizes and demographic information is summarized in Table 2.

**Table 1:** Descriptive characteristics of thalidomide-exposed subjects with compromised hand-function included in this study.

subject	no. of fingers right/left	self-reported foot use
T1-1	4/4	none
T1-2	4/4	none
T1-3	4/4	none
T1-4	4/4	none
T1-5	4/3	none
T1-6	4/3	none
T1-7	4/3	none
T2-1	3/2	occasional
T2-2	3/3	occasional
T2-3	4/3	occasional
T2-4	4/4	occasional
T2-5	4/4	occasional
T2-6	4/4	occasional
T2-7	3/3	occasional
T2-8	3/2	occasional
T2-9	3/3	occasional
T2-10	2/3	occasional
T3-1	0/0	very frequent
T3-2	2/1	very frequent
T3-3	0/1	very frequent

Please note, that self-reported foot use served as main characteristic for group assignment (T1–T3). However, foot use was at least partially determined by the degree of the malformation.

**Table 2:** Group-size with sex-ratio in brackets and age-range (years.) with means in brackets for groups T1–T3, TC and C.

	N (m:f)	age-range (average)
T1	7 (4∶3)	42–44 (43)
T2	10 (6∶4)	39–42 (39.7)
T3	3 (0∶3)	39–40 (39.67)
TC	10 (4∶6)	40–46 (40.7)
C	10 (4∶6)	30–46 (37.9)

With respect to their age, the thalidomide-exposed subjects were very homogeneous as all subjects were born within five years. For most subjects MRI scans of the brain had been acquired in the context of our previous studies [10]–[13]. One subject of group TC and all subjects of group T1 were scanned for the purpose of this morphological study after the other experiments had been completed. T1-subjects were therefore slightly older on average when the scan was taken.

### Imaging and Data Analysis

Structural brain images were acquired on a Siemens Vision 1.5 T scanner (Erlangen, Germany). The T1-weighted data set had a spatial resolution of 1×1×1 mm (TR = 40 ms, TE = 5 ms, flip angle = 40°).

The data were analysed with FSL-VBM, a voxel-based morphometry style analysis [17], [18] carried out with FSL tools [19]. First, structural images were brain-extracted using BET [20]. Next, tissue-type segmentation was carried out using FAST4 [21]. The resulting grey matter partial volume images were then aligned to MNI152 standard space using the affine registration tool FLIRT [22], [23], followed by non-linear registration using FNIRT [24], [25], which uses a b-spline representation of the registration warp field [26]. Images from three randomly selected subjects per group (total of 15 images) were averaged to create a bias-free, study-specific template to which the native grey matter images were non-linearly re-registered.

The registered partial volume images were then modulated (to correct for local expansion or contraction) by dividing by the Jacobian of the warp field. The modulated segmented images were then smoothed with an isotropic Gaussian kernel with a sigma of 4 mm. Finally, voxelwise GLM was applied using permutation-based non-parametric testing with 5000 permutations per contrast. Statistical inference for between subject comparisons was based on threshold-free cluster enhancement [27].

A sensorimotor region of interest (ROI) was created using the Juelich Histological Atlas. This atlas is based on cytoarchitectonic probability maps derived from 10 post-mortem brains [28]. The following Brodmann areas (BA) were included in the ROI: BA 6 [29], BA 4a and BA 4p [30], BA 3a, 3b, and 1 [31] and BA 2 [32]. The probability maps were added and then thresholded at 50% and binarized. By adding the probabilities across maps any voxels included in the ROI had a probability of 50% of belonging to one of the areas of interest. The ROI was finally masked with the study-specific grey matter template mask.

Any differences across the groups within the ROI with p<0.05, corrected for multiple comparisons across space, were accepted as being significant. A more precise localization of significant differences within the sensorimotor ROI was based on the maximum probability maps provided by the Juelich Histological Atlas [28].

To rule out any confound due to different scanning times, we created another template based on the 8 subjects scanned at a later time point (group T1 and one TC subject) and 8 randomly chosen subjects scanned earlier. The native grey matter images were again non-linearly re-registered to this new template. The registered partial volume images were modulated by dividing by the Jacobian of the warp field and smoothed with an isotropic Gaussian kernel with a sigma of 4 mm as described above. Voxelwise GLM was applied using permutation-based non-parametric testing with 5000 permutations for two contrasts comparing “old” and “more recent” scans. For this analysis we used a ROI covering the visual cortex. The ROI included voxels with a summed probability of at least 50% to belong to visual areas V1, V2 or V5 based on the Juelich cytoarchitectonic probability maps [28]. The visual cortex was chosen because we did not expect any differences in this area related to our variables of interest (thalidomide-exposure, compromised hand-function and foot-use). There were significant differences (p<0.05, uncorrected) in only 3% of the voxels, which lies within the limits to be expected for an uncorrected analysis of this threshold. Because there was no evidence to support the notion that different times of scanning were a confounding factor, scans obtained at different time points were analysed together as outlined above.

## Results

There was no main effect of thalidomide within the ROI when all thirty thalidomide-exposed subjects were contrasted with the unexposed control group [T1, T2, T3, TC vs C] and vice versa. Likewise, there were no significant differences within sensorimotor areas when only subjects with normal upper extremities were contrasted [TC vs C].

There was no evidence for *decreased* grey matter values in lateral sensorimotor cortex when subjects with restricted hand function were compared to the control groups with normal hand function [TC, C vs. T1, T2, T3]. To rule out that an effect of malformed upper-extremities was obscured by the effect of hand-like foot use on lateral sensorimotor cortex [13], we excluded T3-subjects and contrasted groups T1 and T2 (no or only occasional foot use) both as a joined group and separately with the control subjects [TC, C vs T1,T2; TC,C vs T1; TC,C vs T2]. There was again no significant grey matter decrease throughout the ROI for any of these contrasts.

Subjects with upper-extremity malformations had been categorised in three groups depending on their different patterns of foot-use (none, occasional, frequent and hand-like). Because this foot-use based categorisation might be less appropriate for the investigation of changes related to the reduced number of fingers, we finally correlated the degree of the malformation (represented by the number of fingers) with grey matter values across all thalidomide-damaged subjects (TC, T1–T3). As the number of fingers was not always strictly symmetrical, we correlated the number of fingers on the right with the left hemisphere ROI and the number of fingers on the left with the right hemisphere ROI. There was no significant positive correlation of number of fingers and grey matter values for either hemisphere, down to a liberal p<0.05, uncorrected.

**Figure 2:**
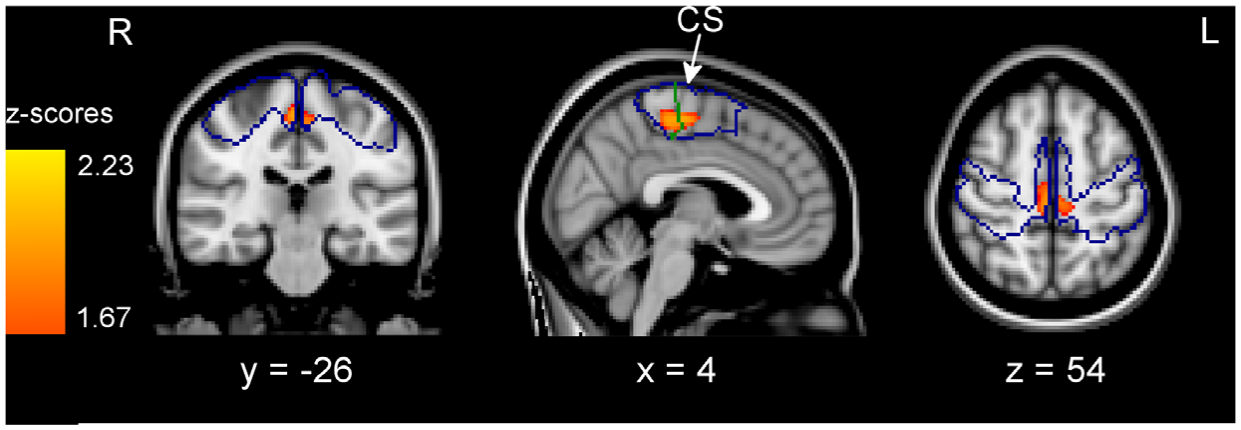
Grey matter increase related to occasional foot use. Areas within the sensorimotor ROI (outlined in blue) showing an increase (p<0.05, uncorrected) in grey matter values for subjects who occasionally use their feet for fine-motor skills when compared to control subjects [T2 vs TC,C]. The green line marks the border between cytoarchitectonic areas BA4 and BA6 based on the Juelich maximum probability maps. Clusters of significant differences are overlaid on the T1-weighted single-subject MNI standard template.

When looking for grey matter *increases* in the dysmelic subjects by reversing the contrast [T1, T2, T3 vs. TC, C] we found significantly higher grey matter values in left medial premotor and sensorimotor cortex. To investigate whether this effect was dependent on the amount of foot use, groups T1–T3 were separately contrasted with the control groups [TC, C]. These contrasts showed no significant increases for group T1 in foot-specific areas. For [T2 vs TC, C] there was a lower threshold (p<0.05, uncorrected) increase of grey matter values restricted to medial parts of bilateral M1 and right supplementary motor cortex (SMA). Localisation of peaks in M1 (x, y, z = 2, −24, 58 and −6, −26, 52, respectively) and SMA (8, −16, 58) was confirmed by Juelich cytoarchitectonic probability maps (Figure 2).

**Figure 3:**
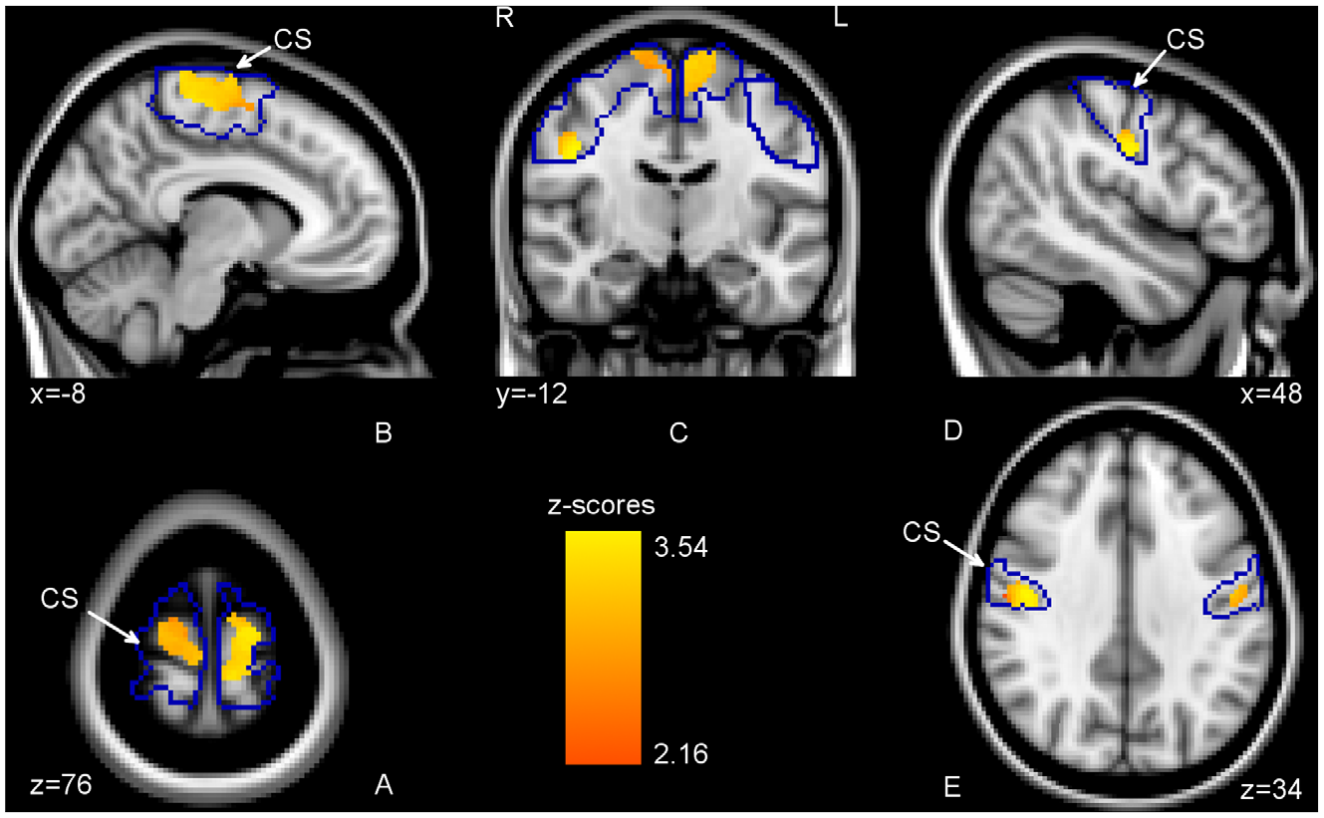
Grey matter increases related to frequent, hand-like foot use. Areas within the sensorimotor ROI (outlined in blue) showing an increase in grey matter values for group T3 when compared to the control subjects [T3 vs TC,C]. Clusters of significant differences are overlaid on the T1-weighted single-subject MNI standard template. (A–D) p<0.05, corrected, and (E) p<0.15, corrected. In T3 subjects, using their feet in a bipedal fashion, increases were more pronounced for the dominant (left) hemisphere as might have been expected from their overall preference for the right foot.

Only subjects with frequent, hand-like everyday foot use (T3) showed significantly increased grey matter values in the medial premotor, dorsolateral premotor, and sensorimotor cortex bilaterally and in the right lateral sensorimotor cortex well underneath the anatomical hand area with p<0.05, corrected (Figure.3 A, B, C, D, Table 3). At a lower threshold, the increase in grey matter values of lateral sensorimotor cortex was seen in both hemispheres (p<0.15, corrected, Figure 3 E).

**Table 3:** Localization of peak differences, together with Z- and uncorrected p-values, for contrasts showing significant (p<0.05, corrected) grey matter value increases.

	[T1,T2,T3 vs TC,C]					[T3 vs TC,C]					[T3 vs T2]				
	x	y	z	Z	p uncorr	x	y	z	Z	p uncorr	x	y	z	Z	p uncorr
PMA L	−2	10	60	2.48	0.007	−10	8	72	2.77	0.003	−10	8	72	2.88	0.002
	−10	2	54	2.99	0.001	−4	12	54	2.75	0.003					
PMA R	8	14	70	3.24	0.0006	8	14	70	3.24	0.0006	12	2	74	2.85	0.002
	6	4	54	3.09	0.001	8	6	54	3.35	0.0004					
M1 L	−10	−30	78	2.99	0.001	−2	−30	62	3.24	0.0006	−10	−34	78	2.99	0.001
M1 R	6	−30	56	3.24	0.0006	4	−30	60	3.54	0.0002	6	−26	78	2.49	0.007
S1 lateral L	−64	−8	32	2.73	0.003	−48	−14	38	2.85	0.002	−58	−6	38	2.69	0.004
S1 lateral R	48	−16	34	2.95	0.002	46	−10	32	3.35	0.0004	46	−14	30	2.91	0.002

To illustrate the similarity between the three contrasts, peaks down to p<0.001, uncorrected, were included. Functional labels are based on highest probabilities derived from the Juelich Histological Atlas.

**Figure 4:**
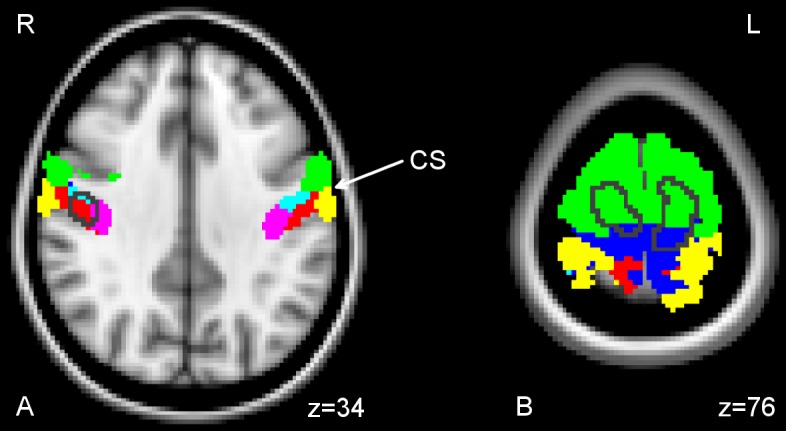
Cytoarchitectonic specification of group differences. This figure shows the spatial relationship of areas with foot use related increases in grey matter values and the cytoarchitectonic anatomy of sensorimotor cortex. The outline of areas showing increased grey matter values for group T3 were overlaid on cytoarchitectonic maximum probability maps. Together they are displayed on the T1-weighted single-subject MNI standard template in axial planes. Green: BA6, blue: BA4a, light blue: BA4p, pink: BA3a, red: BA3b, yellow: BA1, white: BA2. (A) Lateral cluster, (B) bilateral medial cluster. (A) and (B) are based on the [T3 vs TC,C] contrast, thresholded at p<0.05, corrected.

To further specify the localization within the sensorimotor cortex, we evaluated the spatial overlap with the maximum probability maps. The medial cluster of grey matter increase showed most overlap with the maximum probability (MP) maps of premotor (BA 6, 80%) and primary motor cortex (BA 4a, 15%, Figure 4A). There was only small overlap with BA 4p (0.5%), BA 3b (1%), and BA 1 (3.5%). Significant differences in BA6 encompassed both dorsolateral areas of premotor cortex (PMC) and medial areas of SMA.

The overlap of the right lateral cluster was most pronounced in BA 3b (63%), BA 3a (15%) and BA 4p (22%) (Figure 4B).

The (uncorrected) clusters found for [T2 vs TC,C] and significant clusters of difference for [T3 vs TC,C] showed no overlap, the former being localized inferior to the latter. This supports the categorical instead of purely quantitative distinction between groups T2 and T3. To establish whether the increases in grey matter seen in T3 were specific to this group we directly compared T3 with T2 [T3 vs T2]. For this contrast grey matter values in medial and right lateral areas were still significantly increased (p<0.05, corrected). There was substantial overlap with the clusters of difference identified by the [T3 vs TC,C] contrast. At p<0.05, corrected, [T3 vs T2] showed smaller clusters within the increases established by the former contrast and a close to similar pattern at a more liberal p<0.1, corrected. Both contrasts as well as the initial [T1–T3 vs TC,C] contrast showed peak differences in the premotor area (PMA), medial M1, and lateral S1 areas bilaterally with p<0.001, uncorrected (Table 3).

## Discussion

In the present study we determined whether functional representational changes in sensorimotor cortex in subjects with congenitally malformed upper extremities and compensatory skilful foot use [10]–[13] would be paralleled by macroscopic structural changes. Accordingly, we expected decreased grey matter values in lateral sensorimotor cortex related to compromised hand function and increased grey matter values in medial sensorimotor cortex due to compensatory foot use. Consistent with our hypothesis, we found that grey matter values in medial motor areas were increased in subjects with frequent, hand-like (T3) foot use when compared to controls (TC, C). Subjects with occasional (T2) foot use showed subthreshold (p<0.05, uncorrected) grey matter increases in non-overlapping areas inferior to the medial changes seen in T3 subjects but also encompassing parts of SMA and M1, bilaterally. No medial grey matter increases were seen for subjects with malformations but no compensatory foot use (T1). Because there was no effect of exposure to thalidomide on grey matter values, increases in grey matter cannot be attributed to intra-uterine thalidomide exposure in general. Further, grey matter increases in groups T2 and T3 cannot be explained by delayed acquisition of brain scans in T1-subjects. Although the small number of subjects (n = 3 in T3) represents a limitation of this study, the tendency of grey matter increases seen in T2 subjects (n = 10) supports the general notion of foot use related changes. Further, the size and consistency of this effect is also supported by the fact that increases in grey matter values in group T3 reached statistical significance despite the low number of subjects (n = 3).

It is interesting to note that medial grey matter increases were seen bilaterally in both groups. While bilateral changes were expected for T3 subjects with bilateral skilful use of their feet, T2 subjects used their dominant right foot only. We can only speculate about this findings but it may suggest that these movements are supported by both hemispheres as demonstrated for skilful movements of the upper extremities [33]–[35].

Given the design of our study, we were not able to distinguish unambiguously between the effect of frequent, hand-like foot use and severe malformation of the upper extremities with associated compromised hand function. However, considering the fact that size *and* function of upper-extremities were *reduced* in T3-subjects the *increased* grey matter values in medial and lateral sensorimotor cortex seem unlikely to result directly from the upper extremity malformation. Furthermore, the proximity of the medial cluster to the classical area of sensorimotor foot representation strongly suggests a link to frequent and skilful foot use. Finally, T2-subjects with marked malformations and functional restrictions of upper extremities combined with occasional compensatory foot use showed subthreshold evidence for medial grey matter increases in M1 while no changes were detected for group T1, similarly damaged but without compensatory foot use.

Interestingly, subthreshold changes seen for group T2 did not overlap with the more extensive significant changes seen in T3 subjects. This supports the notion of categorical rather than purely qualitative differences between both groups, which guided us when grouping the subjects rather than choosing a parametric approach. As mentioned above, T2 and T3 subjects differed in several ways. Taking furthermore into consideration that the increases in T2 subjects were only found on the uncorrected level, an interpretation of non-overlapping grey matter increases across groups has to remain tentative. However, considering the marked malformations/absence of the upper extremities and the related lack of hand function in T3 subjects, an altered somatotopic organization of sensorimotor areas in these subjects is possible. Along this line we cautiously suggest that the more inferior localization of the (subthreshold) T2 grey matter increases might be due to an inferior-to-superior and medial-to-lateral shift of sensorimotor representations in T3 subjects.

Taken together these results suggest that indeed increased skilful foot use is associated with increases in grey matter of medial M1 and PMA, in line with the increases in the functional foot motor representation observed previously [10], [13].

For the lateral cluster, however, a direct relation to foot use is far less clear. Our T3-subjects had demonstrated an additional functional foot representation in lateral M1, indicating a unique, non-somatotopically organized M1, where the foot was not only represented in the classical medial foot area of M1, but also several centimetres away in non-adjacent cortex in the vicinity of the lateral ‘hand’ area. Both areas had direct output to the spinal motor neurons innervating foot muscles and were behaviourally relevant [13]. However, as hand function was very limited or absent in T3- subjects, no lateral grey matter *increase* was expected as a cause of this additional lateral foot representation in M1. The applied techniques cannot distinguish grey matter related to foot vs hand representation. Furthermore, severe dysmelia/amelia of upper limbs did not allow for a systematic investigation of upper limb sensorimotor representation in group T3. However, for group T2 we demonstrated a *shrinkage* of the functional hand representation in lateral S1 that was related to the number of missing fingers [11], [12]. Electrical stimulation of the feet of T3-subjects did not evoke additional lateral activation in somatosensory areas. Therefore, it is unlikely that the lateral increase in grey matter was related to an abnormal lateral representation of the foot in S1.

Further, and perhaps most importantly, the lateral cluster of grey matter increase reported in the present study was localized well below the classical hand knob [36], corresponding to the classical representation of the face. Although we did not functionally map the entire sensorimotor cortex in our subjects, we hold it unlikely that this area contains either foot or hand representation. This notion is supported by our earlier brain activation studies demonstrating that either movement or electrical stimulation of toes and residual digits of T3-subjects results in activation in the classical foot and hand areas (partly unpublished data). The additional lateral toe movement related activation was clearly located in the vicinity of the hand knob as well. Moreover, stimulation of lateral M1 using TMS elicited motor evoked responses in feet and finger/shoulder muscles simultaneously, indicating a close proximity of lateral foot and hand representation. Taken together, the lack of grey matter increases around the anatomical hand knob thus suggests that indeed neuronal tissue contained in the hand knob and its vicinity is sufficient to house the representation of the remaining hand and the additional lateral motor representation of the foot without significant increases in local grey matter.

It is conceivable that the lateral area of grey matter increase is indeed related to the face representation. We observed a frequent use of the face - in particular the mouth - for object manipulation in our T3-subjects. Unfortunately, this aspect was not formally assessed. Neither have we investigated the face representation in general or the mouth representation in particular in these subjects in any of our previous studies. Thus, any interpretation of the lateral grey matter increase along this line has to remain tentative.

On the basis of cytoarchitectonic probability maps, a large portion (80%) of the medial grey matter increases in T3 subjects was assigned to BA6, mirroring the ratio of PMA and M1 areas size in our ROI. Increases of grey matter were located in both dorsal PMC and medial SMA. A pronounced somatotopic organisation of SMA has recently been demonstrated based on resting state functional MRI data [37]. However, because of the lack of clear anatomical landmarks – such as the hand knob in lateral M1 - a specificity of SMA changes for the foot is hard to establish. Likewise, we cannot rule out that changes in PMC are of a more general nature related to both increased demands for foot movement control and other unusual compensatory motor behaviour not documented by our study, such as chin-shoulder interactions or object control by the mouth.

Of the lateral grey matter increases 78% were assigned to S1 and 22% to M1. This is consistent with the notion that changes in motor cortex are correlated with changes in somatosensory areas and vice versa as might be assumed. However, this assumption contrasts with our findings for the medial area of grey matter increase for which overlap with somatosensory areas was only marginal (1% with BA 3b, 3.5% with BA 1). The use of probability maps may be problematic in this highly specific sample of subjects with altered development of sensorimotor representations due to congenital gross malformations and highly developed compensation from early childhood on.

Another discrepancy was found between functional and structural changes. While we found significant increases in medial grey matter values in group T3 in line with enlarged functional representations [10], [13] we failed to demonstrate grey matter decreases in lateral SM1 for subjects with restricted hand function, apparently contradicting the functional shrinkage demonstrated earlier [11], [12]. This negative result was unlikely to result from small sample sizes alone, as the positive VBM result was obtained with n = 3 (T3) while the negative result was based on n = 30 in the correlation approach and n = 17 (T1, T2) in the categorical contrasts. More likely, our failure to show reduced lateral grey matter suggests that functional representations may shrink without any obvious macroscopic grey matter correlate. Enlarged or reduced functional representations may be paired with more subtle structural changes on the cellular level which might require more fine-grained methods than our non-invasive in-vivo VBM methods presently employed in humans.

Based on self-reports and in accordance with systematic and detailed documentations [38], subjects used their feet spontaneously for grasping and other early motor behaviour from early childhood on, at a time where the sensorimotor system is still developing. For rodent S1 Feldman and Brecht described, how both genetic information and neuronal activity orchestrate the development of maps [2]. It is conceivable that some changes in sensorimotor representation – due to frequent, highly skilled foot use or malformed upper extremities with reduced hand function in this study – are accommodated by purely functional shifts within a macroscopically normal sized S1/M1. More precisely, the somatosensory foot representation might be enlarged by an increased recruitment of silent neurons into the active network [1]. As we did not map other body parts, we cannot determine whether indeed, neighbouring areas show shrinkage/enlargement [39–41].

In this study, we demonstrated that hand-like compensatory foot use was associated with macroscopic grey matter increases in medial motor and premotor areas. These increases paralleled the expansion of functional foot-representations in the same sample of subjects. For medial somatosensory cortex evidence for an expansion of sensory representations was only seen on the functional but not on the structural level. Furthermore, we were not able to demonstrate decreases of grey matter for the lateral hand area in subjects with malformed hands, restricted hand-function and a reduced functional hand representation. Our current techniques might be to coarse leaving us with false negative results, and until now, evidence for adaptive sensorimotor changes are rarely available at the behavioural, functional, and structural level simultaneously. Further research is needed to improve our understanding of how exactly these three levels are interrelated in sustaining lifelong learning and plasticity.
